# Editorial: The role of cofactors in protein stability and homeostasis: Focus on human metabolism

**DOI:** 10.3389/fmolb.2023.1147451

**Published:** 2023-01-25

**Authors:** Marialaura Marchetti, Rita Puglisi, Barbara Cellini, Mirco Dindo, Francesco Marchesani

**Affiliations:** ^1^ Department of Medicine and Surgery, University of Parma, Parma, Italy; ^2^ Randall Centre for Cell and Molecular Biophysics, King’s College London, London, United Kingdom; ^3^ Department of Medicine and Surgery, University of Perugia, Perugia, Italy; ^4^ Okinawa Institute of Science and Technology Graduate University, Okinawa, Japan

**Keywords:** cofactors, F-ATP synthase, cryptochromes, DNA polymerase β, copper trafficking, protein stability and folding

Proteins play a broad range of crucial functions, from extracellular recognition and adhesion to signal transmission, metabolic reactions and expression modulation. In one-third of the cases, their functioning depends on the presence of cofactors. These chemical entities—ranging from simple metal ions to complex organic molecules—can have pleiotropic roles for their target protein, through a combination of structural and functional effects. Indeed, although in some cases cofactors merely contribute to a protein function (such as coenzymes in catalysis), numerous pieces of evidence suggest that cofactors can play a role wider than previously thought, by governing the stability and plasticity of protein tertiary and/or quaternary structures, as well as the ability to properly fold and the intracellular stability of the target protein.

This Research Topic hosts four publications—two original research articles and two mini-reviews—that explore the role of adenine triphosphate (ATP), flavin adenine-dinucleotide (FAD), magnesium, and copper ions cofactors in different physiological contexts, highlighting their key function in regulating specific processes in human metabolism and homeostasis.

Human F-ATP synthase, found in the mitochondrial inner membrane, is the enzyme responsible for ATP synthesis in the final step of the oxidative phosphorylation. The publication authored by Turina reviews the state of the art of F-type ATP synthase regulation, focusing on the possible existence of two different enzyme conformations in charge of ATP and ADP high-affinity catalytic site occupancy. It is conceivable that these two different structural conformations couple the hydrolytic/synthetic enzymatic activity with different H^+^/ATP coupling ratios. Functional and structural pieces of evidence are here reported supporting this putative fascinating regulatory effect, which still waits to be confirmed.

Another intriguing cofactor-related overview presented by Calloni and Vabulas focuses on the FAD role in mammalian cryptochromes. Cryptochromes are transcriptional repressors of circadian genes that regulate circadian rhythms and are evolutionarily related to DNA photolyases. Interestingly, mammalian cryptochromes bind FAD very weakly with KDs above the intracellular concentration of FAD, raising questions about the presence of oscillating local concentrations of cofactor and their putative significance to cryptochromes’ stability. The authors pointed out the scarcity of biophysical data obtained from cellular experiments, that could shed light on the existence of cryptochromes interactors and their role in FAD binding.

The computational research of Srivastava et al. shows the central role of magnesium ions in the activation of DNA polymerase *β*, a DNA-repairing enzyme active especially against oxidative stress damages. Through extensive molecular dynamics simulations, the authors demonstrate that, when the phosphorylation of Ser44 occurs, the enzyme undergoes a conformational rearrangement including the formation of H-bonds and salt bridges; Mg^2+^ ions confer structural flexibility to the polymerase by influencing the salt bridges net, reversing its inactivation upon phosphorylation.

Copper is an essential micronutrient that acts as a cofactor in many important enzymes involved in oxidative phosphorylation, neurotransmitters and melanin synthesis, antioxidant response, iron transport, and connective tissue formation. The copper intracellular concentration must be finely controlled: low metal levels associate with metabolic defects, whereas high levels are cytotoxic. Here, Qasem et al. show that the alteration of copper homeostasis can be exploited as a potential anticancer therapy: metal trafficking can be impaired by using small peptides able to mimic the interaction of copper with its chaperones and transporters. This strategy is an interesting approach to triggering cancer cell death.

Together, these findings show that a deeper understanding of the role of cofactors in protein function and structure is important to unravel the mechanisms behind their activity and regulation, overall stability, and the molecular bases of many metabolic disorders.

